# The effects of high oxygen partial pressure on vegetable *Allium* seeds with a short shelf-life

**DOI:** 10.1007/s00425-020-03398-y

**Published:** 2020-05-16

**Authors:** James E. Hourston, Marta Pérez, Frances Gawthrop, Michael Richards, Tina Steinbrecher, Gerhard Leubner-Metzger

**Affiliations:** 1 Department of Biological Sciences, Royal Holloway University of London, Egham, TW20 0EX UK; 2 Tozer Seeds Ltd, Pyports, Downside Bridge Rd, Cobham, KT11 3EH UK; 3 Laboratory of Growth Regulators, Palacký University and Institute of Experimental Botany, Czech Academy of Sciences, 78371 Olomouc, Czech Republic

**Keywords:** *Allium cepa x Allium fistulosum*, Spring onion, Salad onion, Classical seed ageing, Accelerated artificial ageing, Controlled deterioration, Elevated partial pressure of oxygen (EPPO), Seed  longevity, Seed viability

## Abstract

*****Main conclusion***:**

**Storage at an elevated partial pressure of oxygen and classical artificial ageing cause a rapid loss of seed viability of short-lived vegetable seeds.**

**Abstract:**

Prolonging seed longevity during storage is of major importance for gene banks and the horticultural industry. Slowing down biochemical deterioration, including oxygen-dependent deterioration caused by oxidative processes can boost longevity. This can be affected by the seed structure and the oxygen permeability of seed coat layers. Classical artificial seed ageing assays are used to estimate seed 'shelf-life' by mimicking seed ageing via incubating seeds at elevated temperature and elevated relative humidity (causing elevated equilibrium seed moisture content). In this study, we show that seed lots of vegetable *Allium* species are short-lived both during dry storage for several months and in seed ageing assays at elevated seed moisture levels. Micromorphological analysis of the *Allium cepa* x *Allium fistulosum* salad onion seed identified intact seed coat and endosperm layers. *Allium* seeds equilibrated at 70% relative humidity were used to investigate seed ageing at tenfold elevated partial pressure of oxygen (high pO_2_) at room temperature (22 ºC) in comparison to classical artificial ageing at elevated temperature (42 ºC). Our results reveal that 30 days high pO_2_ treatment causes a rapid loss of seed viability which quantitatively corresponded to the seed viability loss observed by ~ 7 days classical artificial ageing. A similar number of normal seedlings develop from the germinating (viable) proportion of seeds in the population. Many long-lived seeds first exhibit a seed vigour loss, evident from a reduced germination speed, preceding the loss in seed viability. In contrast to this, seed ageing of our short-lived *Allium* vegetable seems to be characterised by a rapid loss in seed viability.

## Introduction

Seed longevity, the life span or 'shelf-life' of mature seeds either in the soil (wet-dry cycles), seed bank or in 'air-dry' warehouse storage, is a critical trait which varies considerably between species (Darwin 1855; Probert et al. 2009; Nagel and Börner 2010). The seed longevity trait is important for wild species as it affects the natural regeneration of plants as well as their genebank seed storage (Walters 2015; Colville and Pritchard 2019). For crop species, it is a key seed quality trait underpinning global agriculture and persistence in the soil (Walck et al. 2011; Finch-Savage and Bassel 2016). Considerable variation in seed longevity is evident among the desiccation-tolerant (usually 5–10% seed moisture) species. The trait depends on genotype (cultivar), seed production environment, and seed storage conditions. The deterioration rate of seeds during storage is accelerated by elevated temperature, relative humidity (RH), and oxygen concentration (Pritchard and Dickie 2004; Walters et al. 2005; Groot et al. 2012). Predicting seed longevity is generally achieved experimentally by classical artificial ageing assays which mimic seed deterioration in a relatively short time. These include accelerated ageing assays and controlled deterioration assays which both combine seed incubation at elevated humidity (generating an elevated equilibrium seed moisture content) and elevated temperature (Powell and Matthews 1981; Ellis et al. 1990; Pritchard and Dickie 2004; Ellis and Hong 2006). While it is clear that low ambient humidity and low temperature are hallmarks of optimal storage conditions for desiccation-tolerant seeds, it is a matter of controversial debate to what extent classical artificial ageing assays can be used to reliably predict seed longevity during storage. Further to this for quality testing in the crop seed industry, it is not known if the classical artificial ageing assays (high humidity and high temperature) can be further accelerated by high oxygen partial pressure.

Crop seed quality is compromised by ageing which initially manifests as reduced seed vigour (germination performance), then subsequently as reduced seed viability (seed death). Alongside vigour and viability loss, ageing also reduces a seed’s ability to produce normal seedlings (Groot et al. 2012; Finch-Savage and Bassel 2016; Schausberger et al. 2019). Using the classical artificial ageing assays provided insight into the molecular processes underpinning the loss of seed vigour and viability. Seed ageing during storage is associated with the oxidation of macromolecules (Bailly 2004; Kranner et al. 2010; Sano et al. 2016). Progressive alterations of cell constituents (proteins, lipids, nucleic acids, sugars, etc.) occur by auto-oxidation processes such as Amadori and Maillard reactions, lipid peroxidation or protein carbonylation (e.g., Salama and Pearce 1993; Chen et al. 2013; Nagel et al. 2015; Sano et al. 2016; Schausberger et al. 2019). The ability to repair accumulated oxidative damage during seed imbibition (antioxidant systems, DNA ligases, *O*-methyltransferases), as well as the biochemical, biomechanical and micromorphological properties of the protecting seed and fruit coat layers are important in determining seed quality (Gardarin et al. 2010; Mene-Saffrane et al. 2010; Waterworth et al. 2010; Sano et al. 2016; Steinbrecher and Leubner-Metzger 2017). It is known for many crop species that mechanical damage including cracks of the seed coat through threshing or shrinkage during drying leads to a reduction in seed quality and viability (Mohamed-Yasseen et al. 1994; Pedretti et al. 2017; Salimi and Boelt 2019). The internal oxygen concentration inside a seed depends on the permeability of the seed coats for gases and on the ambient oxygen concentration (Hermann et al. 2007; Borisjuk and Rolletschek 2009; Schwember and Bradford 2011). These considerations are especially important for short-lived vegetable seeds which include onion, leek, parsnip, pepper and lettuce (Boswell et al. 1940; Justice and Bass 1978; Schwember and Bradford 2011; Roberts 2018; Selvi and Saraswathy 2018).

Ambient air contains 21% oxygen which corresponds to an oxygen partial pressure of 0.021 MPa (norm pO_2_). The loss of vigour and viability during seed ageing is accompanied by accumulated oxidative damage which requires oxygen. Consequently, long-term seed storage at low oxygen concentrations (for example through vacuum packaging) combined with cool and dry conditions are best practice (Schwember and Bradford 2011; Groot et al. 2015). Conversely, dry storage of seeds at 'Elevated Partial Pressure of Oxygen' (EPPO; ~ 18 MPa pO_2_) for 7 weeks, accelerated the seed deterioration processes of lettuce, cabbage and barley, resulting in reduced seed vigour, viability, and the percentage of normal seedlings (Groot et al. 2012). Furthermore, dry storage EPPO treatment of barley and Arabidopsis mapping population seeds delivered quantitative trait loci (QTLs) and proved genetically that EPPO mimics and accelerates dry after-ripening storage and ageing (Nagel et al. 2016; Buijs et al. 2018). EPPO with a ~ 850-fold elevated oxygen partial pressure (~ 18 MPa pO_2_) therefore provided a method for analysing seed ageing under dry conditions which is faster (weeks) compared to 'natural' ageing during dry storage (months) in ambient air at norm pO_2_ (Groot et al. 2012). Short-lived vegetable seeds such as onion or lettuce have typically 4–5% moisture content in their dry state (at < 15% RH) and such a low seed moisture content is also best for seed long-term dry storage (Nagel and Börner 2010; Schwember and Bradford 2011; Selvi and Saraswathy 2018). Results from classical artificial ageing assays utilising high relative humidity (70–80% RH) and high temperature (typically 42 °C) have been found to elevate the moisture content of the seeds to typically 9–13%. Whether elevated seed moisture combined with high oxygen partial pressure (elevated pO_2_) provide a method for analysing seed ageing and potentially a fast diagnostic assay for seed quality is not known. In the work, we present here we investigated if elevated seed moisture combined with tenfold elevated pO_2_ compares to classical artificial ageing in assays using very short-lived *Allium* vegetable seeds.

## Materials and methods

### Plant materials

Salad onion seed of the interspecific *Allium cepa* L. x *Allium fistulosum* L. cv. Guardsman was harvested from plants grown in 2014 at the UK company site (trial seed lot S60/342 (hereafter '342′), Tozer Seeds Ltd. Pyports, Downside Bridge Road, Cobham, Surrey, KT11 3EH, UK) and at the seed production site Klein Karoo in South Africa (trial seed lot '016′ and commercial seed). Further to these seed lots, commercial seed of *Allium cepa* cv. Hyfive (bulb onion) and *Allium porrum* cv. Lancaster (leek) was used (Table 1). The 1000 kernel weight of *A. cepa* x *A. fistulosum* seed was determined according to ISTA (International Seed Testing Association) standard protocols, measured by weighing 8 × 100 seeds and extrapolating a mean weight of 1000 seeds. Seed moisture content (SMC, expressed per dry weight) was measured using a moisture analyser (*n* = 4 × 100, HB43-S, Mettler-Toledo Ltd, Leicester, UK), seed water activity was measured with a water activity meter (*n* = 3 × 100, LabMaster-aw, Novasina AG, Lachen, Switzerland).

**Table 1:** Effect of seed dry storage and ageing treatments on the maximum germination percentages (*G*_max_) of *Allium* species seed lots in ambient air

*Allium* species	Cultivar and seed lot	*G*_max_ [%]^1^ before storage	Relative humidity [RH] and temperature (°C)	Storage or treatment duration *G*_max_ [%]^1^ after storage or treatment			References
"Natural" ageing during longer dry storage at room temperature (RT)				ca. 8 months	12 months	21 months	
*A. cepa x A. fistulosum* (Salad onion)	Guardsman lot 342	91.8 ± 2.8	Desiccant at 22 °C	–	72.1 ± 3.1	60.0 ± 4.6	This work
	Guardsman lot 061	93.3 ± 2.0	Desiccant at 22 °C	–	71.1 ± 8.0	–	This work
*A. cepa* (Bulb onion)	Mean of 20 lots	91.5 ± 1.2	Storage at RT	49.3 ± 5.9	–	–	Delouche and Baskin (1973)
	Mean of 8 tests^2^	80.2	66% RH at 27 °C	37.4	–	–	Boswell et al. (1940)
	Mean of 8 tests^2^	80.2	44% RH at 27 °C	72.9	–	–	Boswell et al. (1940)
*A. fistulosum *(Welsh onion)	Zhangqiu	90.6 ± 7.3	45% RH at RT	–	57.0 ± 3.6	–	Dong et al. (2014)
Classical artificial ageing at increased humidity and temperature				4–5 days^5^	7–8 days^6^	10 or 30 days	
*A. cepa*	Hyfive (commercial seed)	94.5 ± 3.7	90% RH at 42 °C	–	28.9 ± 7.8	–	This work
*A. porrum* (Leek)	Lancaster (com. seed)	93.3 ± 1.7	90% RH at 42 °C	53.3 ± 7.3	0.0 ± 0.0	–	This work
*A. cepa x A. fistulosum*	Guardsman (com. seed)	76.7 ± 1.7	90% RH at 42 °C	15.0 ± 2.9	1.7 ± 1.7	–	This work
	Guardsman lot 342^3^	72.1 ± 3.1	70% RH at 42 °C	–	60.0 ± 7.0	50.8 ± 3.5 (10d)	This work
	Guardsman lot 342^4^	70.0 ± 6.6	70% RH at 22 °C	–	–	72.2 ± 5.0 (30d)	This work
*A. cepa*	Mean of 20 lots	91.5 ± 1.2	100% RH at 42 °C	36.0 ± 5.5	–	–	Delouche and Baskin 1973
	Mean of 5 lots	69.6 ± 6.8	94% RH at 42 °C	18.8 ± 4.3	–	–	Madruga de Tunes et al. (2011)
	Mean of 5 lots	69.6 ± 6.8	76% RH at 42 °C	39.6 ± 5.9	–	–	

^1^Mean values ± SE of at least three replicates each with 30 seeds

^2^Mean of 8 × 100-seed tests

^3^Conducted with 12 months stored seeds

^4^Conducted with 24 months stored seeds, *G*_max_ values obtained (at 172 h in the germination kinetics) were not significantly different: 70.0 ± 6.6% (without incubation) compared to 72.2 ± 5.0% (30 days incubation at 70% RH at 22 ℃). Treatment with 10 µM GA_4+7_ resulted in 81.1 ± 3.2% and 78.9 ± 4.7% *G*_max_, respectively (at 365 h in the germination kinetics) which verified that most of the non-germinating seeds prior to the GA treatment were not viable

^5^4 days, except for Delouche and Baskin 1973 (5 days)

^6^7 days for *A. cepa* and Guardsman lot 342, 8 days for *A. porrum* and Guardsman commercial seed

### Seed ageing treatments

**Fig. 1:**
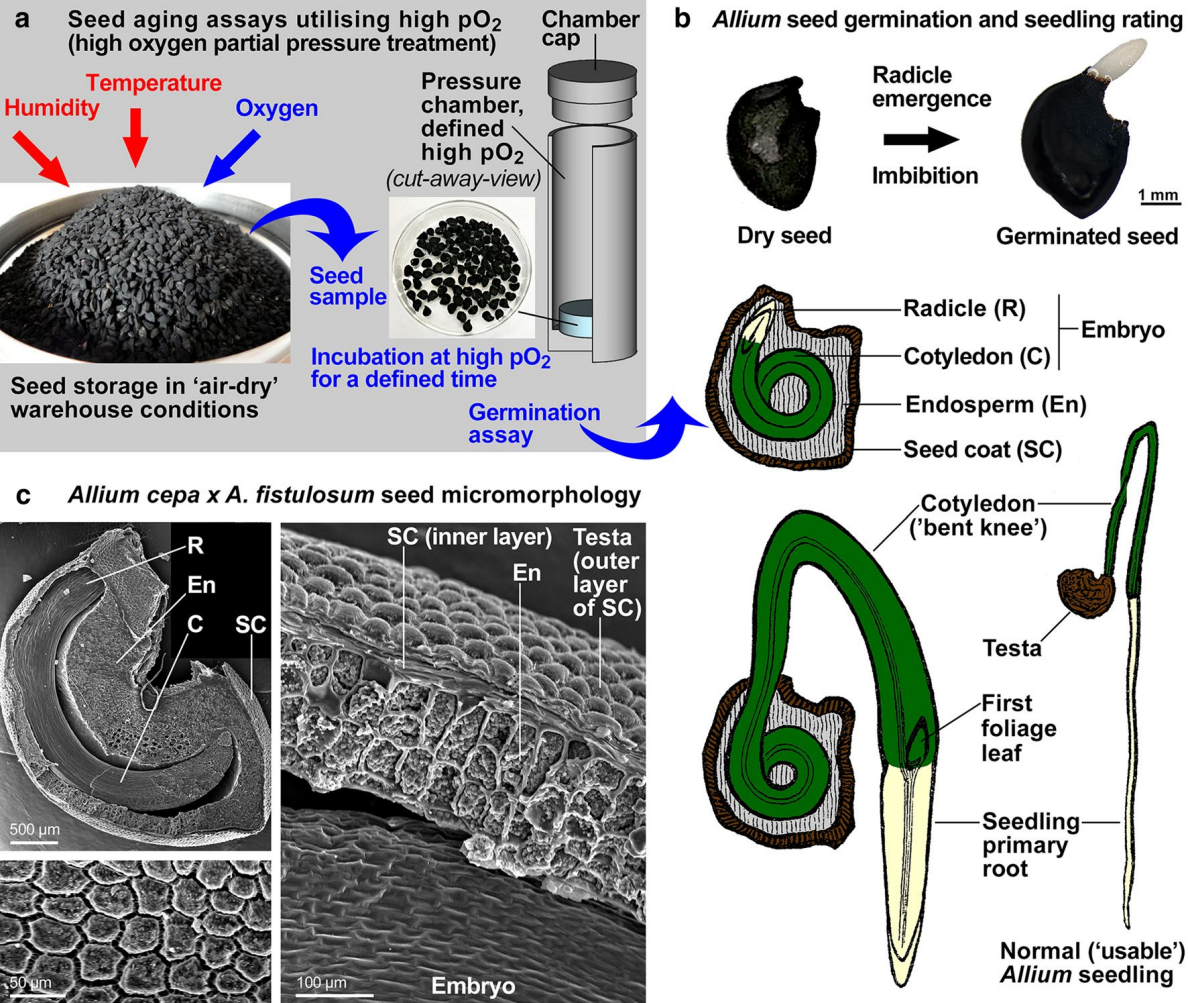
Seed micromorphology, germination and ageing assays for *Allium* species. **a** Seed ageing of *Allium* seed utilising a pressure chamber capable of generating and maintaining a defined elevated ambient oxygen partial pressure (pO_2_). Straight arrows depict the three ambient factors that affect aging and seed quality during storage. **b***Allium* seed and seedling structure and germination. Drawings modified from Julius Sachs (1887) Vorlesungen über Pflanzem-Physiologie, Verlag von Wilhelm Engelmann, Leipzig. **c** Seed morphophysiology of the *Allium cepa x Allium fistulosum* cv. Guardsman analysed by SEM. Left top: Cross section through a mature seed. Left bottom: View onto the outer surface of the testa revealing cell shape and sculpturing; note that this is of taxonomic importance (Celep et al. 2012). Right: Cross section of the seed coat and endosperm layers. *R* radicle, *En* endosperm, *C* cotyledon, *SC* seed coat

A classical artificial ageing treatment was applied by subjecting dry seeds (~ 4% SMC) to 70% relative humidity (RH, in the headspace of a sealed container above 25 g/100 mL LiCl) at 42 °C for 3, 7 and 10 days. These conditions were informed by literature on other *Allium* seed ageing experiments (Salama and Pearce 1993) and further experimental optimisation of the assay conditions. A control for this classical artificial ageing assay was a 10-day treatment at 70% RH at 22 °C. After the treatment, seeds were then dried back to ~ 4% SMC above silica gel prior to germination assays. As a comparison to a high temperature (42 °C) and moisture (70% RH) treatment, we subjected seeds to a high oxygen partial pressure treatment (high pO_2_ ageing assay). Prior to elevated pO_2_ treatment, seeds were equilibrated at 70% RH (at 22 °C and norm pO_2_) for 2 days to ensure that they have elevated SMC (~ 9.5% was measured) as in the classical artificial assay. For the high pO_2_ ageing assay seeds were exposed to compressed air at 0.21 MPa pO_2_ (10 bar compressed air) for 10 and 30 days at room temperature (22 °C) using a high-pressure chamber (3000F01 plant water status console, 0.5 L, Soil Moisture Equipment Corp., Goleta, CA, USA) with a modified chamber cap (Fig. 1a). Compressed air (BOC UN1002 with ~ 250 ppm moisture at 200 bar) will generate 60–70% RH at 10 bar. After the high pO_2_ ageing treatment the seeds were dried back at normal atmospheric pressure to ~ 4% SMC above silica gel. As a control for the 30-day high pO_2_ ageing assay at 22 °C we conducted a 30-day treatment at 70% RH (at 22 °C).

### Scanning electron microscopy (SEM)

Dry, mature *Allium* seeds were mounted on 12.5 mm Cambridge aluminium specimen stubs, sputter-coated with gold (40 nm thickness, Polaron SEM Coating Unit E5100, Bio-Rad Microscience Division, UK) and studied using SEM (Hitachi S-3000 N, Tokyo, Japan) at an acceleration voltage of 20 kV.

### Germination and seedling phenotype assays

Germination assays were conducted under continuous white light (~ 100 µmol s^−1^ m^−2^) at 20 °C (incubator MLR-352-PE, Panasonic, Osaka, Japan). Onion seeds were incubated in 6 cm Petri dishes containing two ⌀ 5 cm filter paper disks (MN713, Macherey–Nagel, Dueren, Germany) and 2 mL dH_2_0 + 0.3% PPM™ (Plant Cell Technology, Washington, DC, USA). Each of 6 replicates consisted of a Petri dish containing 15 onion seeds with germination defined as the radicle protruding through the testa by 1 mm. For seedling phenotype assays onion seeds were incubated at 20 °C in clear plastic boxes (118 mm x 176 mm x 42 mm) containing a sheet of filter paper and a pleated filter paper (Gilson Scientific Ltd, Houghton Regis, UK) each containing 100 onion seeds and 50 mL of dH_2_0. Seedlings were assessed after 14 days. Normal seedlings were defined as having the primary root intact and the cotyledon with a characteristic bent ‘knee’. Abnormal seedlings were typified by a primary root that is stunted and/or displays negative geotropism, cotyledons that are short or undeveloped, a looping or spiral form rather than the presence of a bent ‘knee’. Any one of the features of abnormality would result in a seedling being classified as abnormal as such a seedling would not produce a commercially viable ('usable') plant. Testing for abnormality is commonly practiced throughout the seed industry (Ignatz et al. 2019). The maximal germination percentage (*G*_max_) was calculated from the maximum number of seeds that germinated as a proportion of the total number of seeds and analysed using a generalised linear model with binomial errors using R version 3.6.0 (R Core Team 2019).

## Results and discussion

Seeds of onion (*Allium cepa* L.) and related *Allium* species are considered to be among the shortest lived of all common vegetable crops (Boswell et al. 1940; Justice and Bass 1978; Schwember and Bradford 2011; Roberts 2018; Selvi and Saraswathy 2018). These works classify onion, leek, parsnip, pepper and lettuce as examples of short-lived vegetable seeds of storage category 1 typically characterised by only 1–2 years shelf-live (Justice and Bass 1978; Roberts 2018). Table 1 shows that the maximum germination (*G*_max_) of our salad onion *A. cepa x A. fistulosum* cv. Guardsman seed lot 324 decreased from ~ 92 to ~ 60% during 21 months of dry storage at room temperature. This seed lot had a 1000 seed weight of 5.11 g and the dry seed had a moisture content of 4.2 ± 0.1% corresponding to a water activity of *a*_w_ = 0.33 ± 0.01. That the observed reduction in maximum germination (*G*_max_) is in fact due to a reduction in lot viability by progressing death of individual seeds and not by the induction of secondary physiological dormancy (Finch-Savage and Leubner-Metzger 2006) was verified by gibberellin treatment which demonstrated that most of the non-germinating seeds were indeed dead (Table 1). A rapid reduction in seed lot viability caused by 'natural' ageing during long-term storage in the dry state (4–6% SMC) is likewise evident for other *Allium* species (Table 1). With at least 660 species, the monocotyledon genus *Allium* is one of the largest, harbouring major vegetable crops including *A. cepa* (common or bulb onion), *A. fistulosum* (Welsh or Japanese bunching onion), *A. sativum* (garlic), *A. porrum* (leek), and *A. schoenoprasum* (Brewster 2008; Dong et al. 2014; Hauenschild et al. 2017; Selvi and Saraswathy 2018). A number of hybrids are cultivated as salad or spring onions that have the closely related *A. cepa* and *A. fistulosum* species as parents (Hauenschild et al. 2017; Kudryavtseva et al. 2019). In addition to their economic importance, the very short shelf-life of *Allium* vegetable seeds provide an excellent system to study the mechanisms of seed ageing and to develop diagnostic assays.

In a mature *Allium* seed, the fully developed embryo is embedded in a living endosperm tissue which is surrounded by a dead seed coat (Fig. 1). Radicle emergence was used as visible event to mark the completion of seed germination. Subsequent embryo growth into a normal seedling ('usable' for primary crop production) is characterised by the development of an intact primary root and a cotyledon with a characteristic bent ‘knee’ (Fig. 1b). As for all *Allium* seeds, the *A. cepa x A. fistulosum* outer seed coat layer (testa) is black (Fig. 1b) and the micromorphology of the testa cells (Fig. 1c) is very similar if not identical to *A. cepa* and *A. fistulosum* for which irregular polygonal, loose cell shapes are characteristic (Celep et al. 2012; Choi et al. 2012; Lin and Tan 2017). Between the *A. cepa x A. fistulosum* testa and endosperm layers is a layer of crushed seed coat cells (Fig. 1c) the location and structure of which corresponds to the semipermeable seed coat layer characterised microscopically and biochemically in onion seeds (Beresniewicz et al. 1995a, b, c). Maintaining seed quality during dry seed storage is dependent on the properties of the seed coat which mediates moisture uptake and gas exchange. Mechanical damage is known to reduce the quality of dry onion seeds (Pedretti et al. 2017), but as our micromorphological analysis of dry *A. cepa x A. fistulosum* seeds did not reveal any obvious damage (Fig. 1) we used seed lots of this and other *Allium* species to optimise the conditions for the classical artificial aging assays (Table 1).

**Fig. 2:**
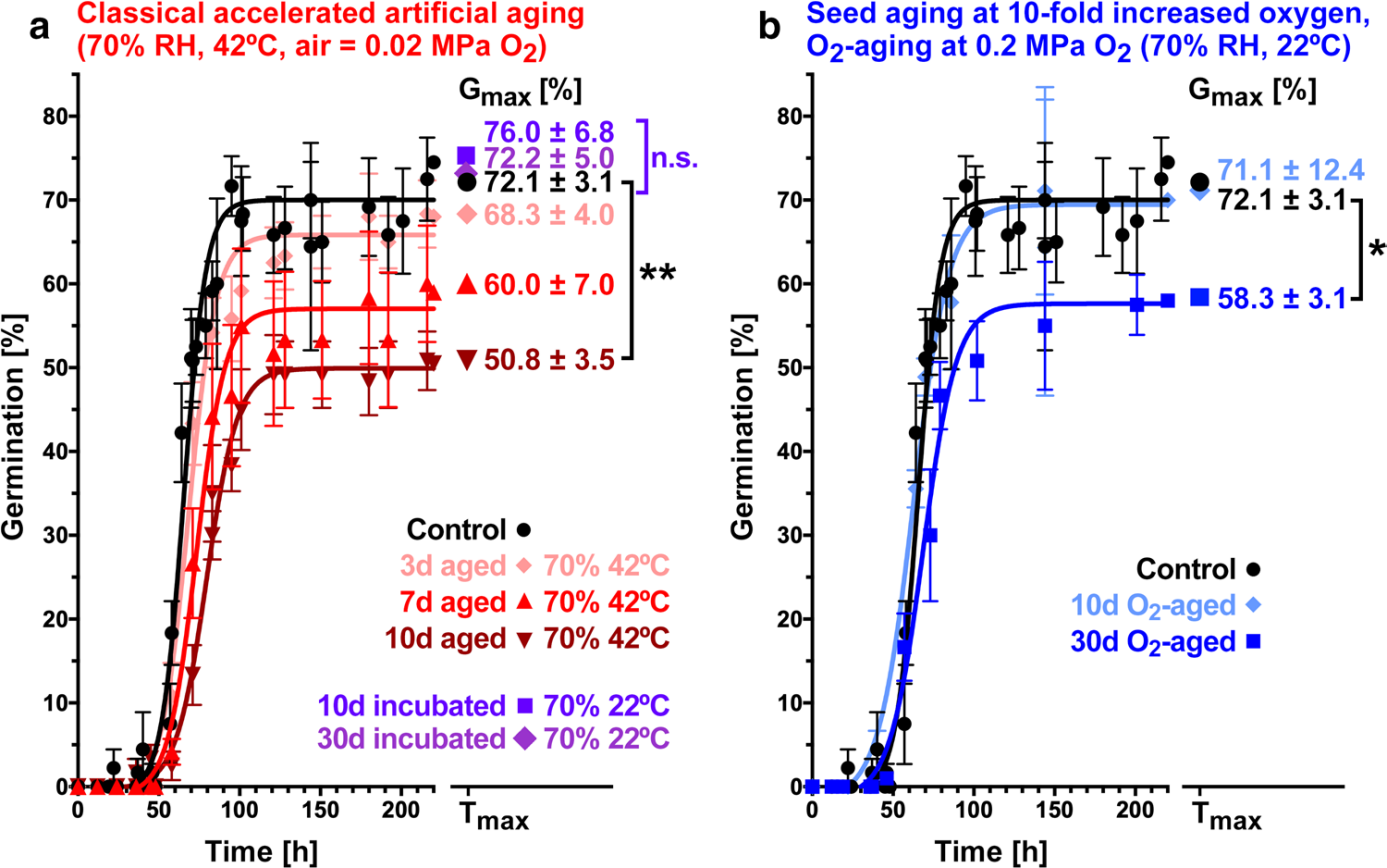
The comparison of seed ageing assays. **a** Classical artificial (70% RH, 42 °C) and **b** high oxygen partial pressure (elevated pO_2_) seed ageing assays of the *Allium cepa x Allium fistulosum* cv. Guardsman. *G*_max_ values shown are means ± standard error. Control treatments include incubation for 10 and 30 days at 70% RH and 22 °C which does not affect the *G*_max_, which demonstrated that the elevated SMC alone does not cause seed viability loss. A 10 µM gibberellin A_4+7_ (GA) treatment does not affect the *G*_max_ and thereby demonstrated that non-germinating seeds are dead: GA-treatment of 30-day elevated pO_2_ aged seeds resulted in a *G*_max_ of 50.8 ± 16.3% which is statistically not different to the not GA-treated seeds. The same conclusion was obtained from GA-treatment of control seeds (see Table 1 for *G*_max_). Statistically significant differences between *G*_max_ values are denoted as *p < 0.05 and **p < 0.01; n.s., not significant. *T*_max_ is the maximum time of the germination assay and is between 225 and 280 h

In both 'naturally' aged (dry stored long-term dry storage ~ 5% SMC, Table 1) and in classical artificial ageing assays at 70–90% RH and 42 °C a rapid decline in seed lot viability was observed for different *Allium* species (Table 1). After 2 days of equilibration at 70% RH our *Allium* 342 seed lot had a 9.5 ± 0.3% SMC; 70–80% RH is known to elevate the *Allium* SMC to ~ 9–13% and 90–100% RH to >15% (Boswell et al. 1940; Salama and Pearce 1993; Madruga de Tunes et al. 2011; Schwember and Bradford 2011; Selvi and Saraswathy 2018). A temperature of 40–50 °C combined with 70–100% RH was found to provide optimal assays for *Allium* seed lots (Boswell et al. 1940; Delouche and Baskin 1973; Justice and Bass 1978; Salama and Pearce 1993; Madruga de Tunes et al. 2011; Schwember and Bradford 2011; Selvi and Saraswathy 2018). A temperature of 42 °C was used in most of these classical artificial ageing assay including in our works (Table 1). Submitting our *A. cepa x A. fistulosum* seed lot 342 to 42 °C combined with 70% RH for 10 days reduced *G*_max_ from 72.1 to 50.8%. In contrast to 42 °C, the *G*_max_ of the control treatment at 22 °C (70% RH, 10 days) was 76.0%, clearly demonstrating that elevated SMC alone is not reducing the seed lot viability (Table 1). Figure 2a shows that our classical artificial ageing assay caused a progressive and rapid viability loss, the severity of which increased with the duration of the treatment. This loss in seed lot viability was not preceded by a visible decline in speed (*T*_50%_ of *G*_max_) or vigour as is known from lettuce and many other species (Schwember and Bradford 2011; Groot et al. 2012; Finch-Savage and Bassel 2016). It rather seems that for individual *Allium* seeds, ageing may trigger a switch towards seed death and hence without an apparent decline in seed lot germination speed causes a rapid decline in seed lot viability (Fig. 2 and Table 1).

To assess how ageing of short-lived *Allium* seeds compares in high pO_2_ versus classical artificial seed ageing assays, we first equilibrated the salad onion (*A. cepa x A. fistulosum* cv Guardsman) seeds at 22 °C in 70% RH to ensure that the same elevated SMC (9.5 ± 0.3%) is reached. The seeds were subsequently incubated either in the classical artificial ageing assay (70% RH, 42 °C) for 3–10 days (Fig. 2a) or in a pressure chamber (Fig. 1a) for the high pO_2_ assay at tenfold elevated pO_2_ (0.21 MPa O_2_, 70% RH, 22 °C) for several days (Fig. 2b). Subsequent analysis of germination data demonstrated that 30 days high pO_2_ treatment significantly reduced (*P* < 0.05) the maximum germination percentage (*G*_max_) from ~ 72 to ~ 58% while 10 days high pO_2_ treatment did not affect the germination response (Fig. 2a). The ~ 14% reduction in *G*_max_ by 30 days high pO_2_ seed ageing was due to loss of seed viability. As for the classical artificial ageing, gibberellin treatment was used to verify that the observed reduction in seed lot *G*_max_ was indeed due to a reduction in seed lot viability (Fig. 2). As in the classical artificial ageing assay (humidity × high temperature 42 °C) the major response of the *Allium* seed population was, therefore, a reduction in *G*_max_ due to loss of seed viability. Compared to the seed ageing responses in the classical artificial ageing assay (Fig. 1a) the 30 days high pO_2_ seed ageing corresponds to approximately 7 days ageing at humidity x high temperature (Fig. 2). No appreciable reduction in germination speed or vigour was evident in the *Allium* seed samples, the major effect in both ageing assays was due to the rapid loss in seed viability of a proportion of seeds in the population. We, therefore, show here that tenfold elevated pO_2_ (0.21 MPa O_2_) caused a rapid loss in seed viability per se at room temperature after 30 days (Fig. 2b). A rapid loss of seed viability in both seed ageing assays for *Allium* suggest that oxidation processes including lipid oxidation is a major mechanism for the oxygen-dependent seed ageing of *Allium* (Salama and Pearce 1993; Bailly 2004; Nagel and Börner 2010; Groot et al. 2012; Sano et al. 2016). A recent study on 'natural' ageing by 5–40 years seed dry storage of wheat and barley demonstrated that also the long-term age-dependent loss of seed viability is associated with increased lipid oxidation (Wiebach et al. 2019). The salad onion (*A. cepa x A. fistulosum* cv. Guardsman) cultivar used in our study represents a very typical commercial *Allium* seed lot based on Tozer's experience as a seed company. That this is indeed the case was confirmed in our work by the expanded analysis of 'natural' and classical artificial ageing across other *Allium* species and seed lots (Table 1). From these results, the observed rapid seed viability loss indeed seems to be a hallmark of *Allium* seed ageing during storage. We observed the rapid loss in seed viability response also after 30 days in the tenfold elevated pO_2_ ageing assay with the specific seed lot used in our experiment (Fig. 2b). Because we obtained a typical *Allium* seed ageing response, namely viability loss, we suppose that this is a more general response for *Allium* seed ageing during storage in any quality assay. Further work with several *Allium* seed lots to analysing the duration and intensities of humidity, temperature, and oxygen as seed ageing assay factors is therefore required and should also be expanded to other species with short seed shelf-life.

**Fig. 3:**
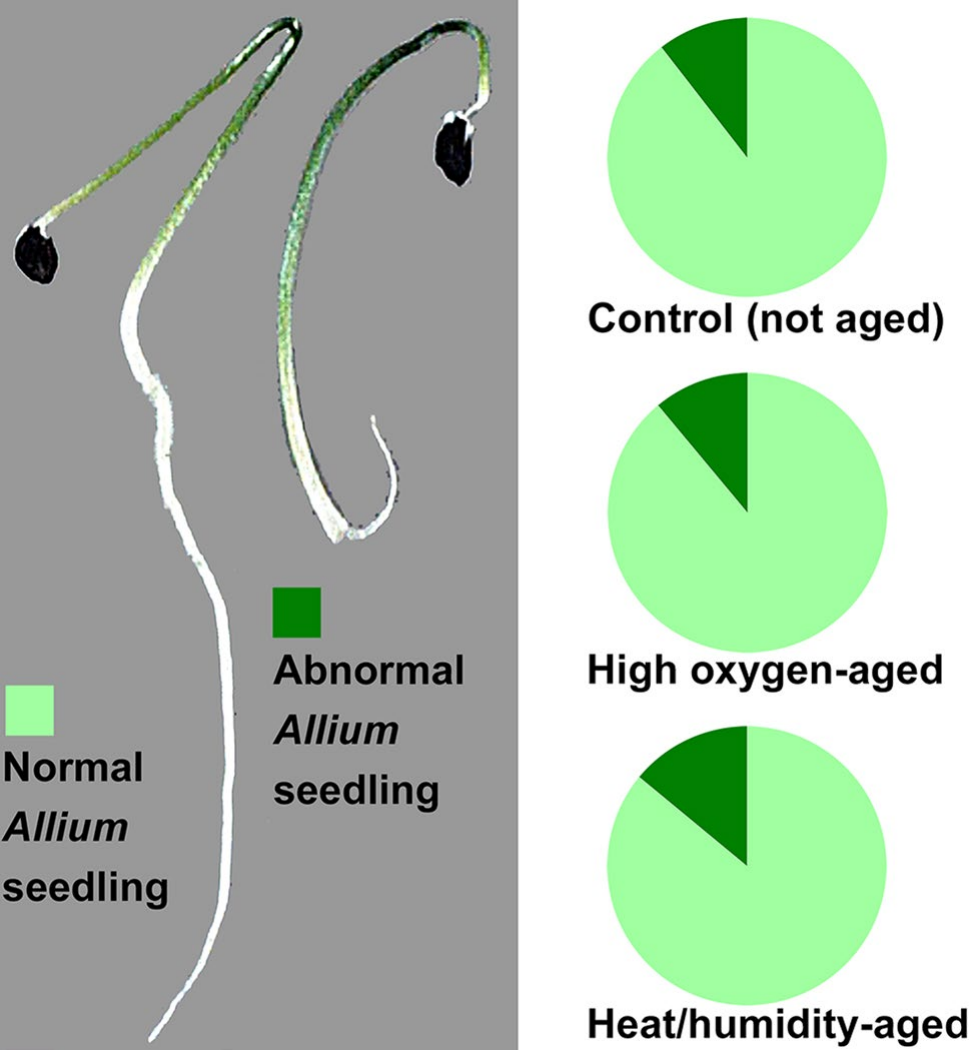
Summary of *Allium cepa x Allium fistulosum* cv. Guardsman seedlings developing from the germinated (viable) seeds of the seed populations, subjected to either 30 days high pO_2_ or 7 days classical artificial seed ageing assay conditions. Left: Effect of ageing on *Allium* seedling phenology. Right: Pie charts presenting the relative proportions of normal and abnormal *Allium* seedlings

A rapid loss of *Allium* maximal seedling emergence in ambient air conditions (0.021 MPa pO_2_) was reported by using classical accelerated ageing assays (Salama and Pearce 1993) and controlled deterioration assays on the percentage of normal seedlings (Schwember and Bradford 2011). These results allow no distinction between seed viability per se and the negative effects of the ageing on seedlings (death/abnormality) from the germinating seeds of the population. Groot et al. (2012) showed that dry storage EPPO treatment of lettuce seeds with 850-fold elevated pO_2_ (18 MPa O_2_) reduced germination speed and maximum germination of the lots. For seed lots stored for at least 3 years in warehouse conditions, it also increased the number of abnormal lettuce seedlings. Normal, usable seedlings are key for the crop seed industry and the dry storage EPPO treatment, therefore, provided a fast method for analysing lettuce seed lot quality during long-term dry storage (Groot et al. 2012). In contrast to this, our results with *Allium* seedlings (Fig. 3) showed that neither the classical nor the high pO_2_ treatment with tenfold elevated pO_2_ (0.21 MPa O_2_) affected the incidence of abnormal seedlings originating from the viable seeds which are germinating. Because it is faster, the optimised classical artificial ageing assay based on elevated RH and temperature is more suited for testing *Allium* seed quality. It seems that *Allium* species are very sensitive to seed ageing and instantly respond with a rapid loss of seed viability while species with a longer 'shelf-life' respond with vigour loss and subsequently with a slow loss of seed viability. The mechanisms and genetics (Nagel et al. 2016; Sano et al. 2016; Buijs et al. 2018; Schausberger et al. 2019) underpinning the distinct sensitivities to dry storage EPPO treatment and high pO_2_ treatment of seeds equilibrated to a higher SMC is a timely topic for future research. It has been proposed from experiments with onion and lettuce comparing seed ageing at low and normal pO_2_ that different molecular mechanisms may be involved in seed ageing at different moisture levels and that norm pO_2_ levels are more harmful to seeds at lower SMC compared to elevated SMC (Ibrahim et al. 1983; Ellis and Hong 2007; Schwember and Bradford 2011). The level of peroxidation was proposed to be an important difference between fast and slow ageing seeds, but the speed of deterioration also depends on the cellular redox environment, changes in pH, storage compounds and genotype (Salama and Pearce 1993; Selvi and Saraswathy 2018; Nagel et al. 2019). Our findings with *Allium* seed ageing at elevated SMC demonstrate that the combination with either elevated pO_2_ or temperature both cause rapid seed viability loss without appreciably affecting germination speed. Short-lived *Allium* vegetable seeds, therefore, provide an excellent system to study the biochemical mechanisms of seed lot viability loss per se using ageing assays in which the distinct contributions of all three ambient cues (humidity, temperature, oxygen) can be investigated individually and in combination.

### *Author contributions Statement*

MP, TS, FG, JEH and GL-M planned and designed the research; JEH, TS and MR performed experiments; MP, FG and MR provided material or equipment; TS, JEH, and GL-M analysed and interpreted the data; JEH and GL-M wrote the manuscript with contributions of all authors.

## Abbreviation

SMC: Seed moisture content
